# Multimodal prediction of psychotic-like experiences using elastic net modeling: external validation in a clinical sample

**DOI:** 10.1017/S0033291725102201

**Published:** 2025-11-14

**Authors:** Seda Arslan, Merve Kaşıkçı, Osman Dağ, Didenur Şahin-Çevik, Işık Batuhan Çakmak, Evangelos Vassos, Martijn van den Heuvel, Timothea Toulopoulou

**Affiliations:** 1 Department of Psychology, Bilkent University Faculty of Economics Administrative and Social Sciences, Ankara, Türkiye; 2 Department of Biostatistics, Hacettepe University, Ankara, Türkiye; 3 Department of Neuroscience, Bilkent University, Ankara, Türkiye; 4 Department of Psychiatry and Psychotherapy, Central Institute of Mental Health, Medical Faculty Mannheim, Heidelberg University, Mannheim, Germany; 5 Department of Psychiatry, University of Health Sciences, Ankara Bilkent City Hospital, Ankara, Türkiye; 6 Social, Genetic and Developmental Psychiatry Centre, Institute of Psychiatry, Psychology and Neuroscience, King’s College London, London, UK; 7 National Institute for Health and Care Research (NIHR) Maudsley Biomedical Research Centre (BRC), London, UK; 8 Department of Complex Trait Genetics, Center for Neurogenomics and Cognitive Research, Vrije Universiteit Amsterdam, Amsterdam, Netherlands; 9 Department of Child and Adolescent Psychiatry, Section Complex Trait Genetics, Amsterdam Neuroscience, Vrije Universiteit Medical Center, Amsterdam UMC, The Amsterdam, Amsterdam, Netherlands; 10 Department of Psychology, Department of Neuroscience, İhsan Doğramacı Bilkent Üniversitesi: Bilkent Universitesi, Ankara, Türkiye; 11 Department of Psychiatry, National and Kapodistrian University of Athens, Athens, Greece; 12 Department of Psychiatry, Icahn School of Medicine at Mount Sinai, New York, NY, USA

**Keywords:** elastic net modeling, machine learning, psychotic-like experiences, psychosis first episode, structural connectome

## Abstract

**Background:**

Psychotic-like experiences (PLEs) are considered a subclinical component of psychosis continuum. Studies indicate that PLEs arise from multimodal factors, yet research comprehensively examining these factors together remains scarce. Using a large youth sample, we present the first model that simultaneously examines multimodal factors related to PLEs. As a secondary aim, we evaluate the model’s ability to explain psychosis in an external validation cohort that included individuals experiencing psychosis.

**Methods:**

After applying variable selection including generalized estimating equations, correlation filtering, Least Absolute Shrinkage and Selection Operator model to 741 variables (i.e., environmental factors, cognitive appraisals, clinical variables, cognitive functioning, and structural brain connectome measures), obtained PLEs predictors (*N* = 27) and covariates (i.e., age, sex, IQ) were included in the classification model based on Elastic Net algorithm for predicting high/low PLEs in 396 healthy participants aged 14–24 (*M_age_* = 19.72 ± 2.5). We externally validated PLE-related predictors in a clinical sample comprising first-episode psychosis patients (*n* = 19), their siblings (*n* = 20), and healthy controls (*n* = 19).

**Results:**

Eleven factors, including environmental and cognitive appraisals, along with 16 structural network properties spanning frontal, temporal, occipital, and parietal regions, were identified as important predictors of PLEs. The model’s performance was moderate in predicting low versus high PLEs (accuracy = 75%, AUC = 0.750). Specificity was high (84.2%) in distinguishing siblings from patients.

**Conclusions:**

Multimodal features, including environmental burden, cognitive schemas, and brain network alterations, predict PLEs and partially generalize to clinical psychosis. These variables may reflect intermediate phenotypes across the psychosis spectrum, offering insights into both vulnerability and resilience.

## Introduction

Psychotic experiences, including hallucinations and delusions, are most commonly associated with schizophrenia but are also reported across other psychiatric disorders (Bourgin et al., 2020; Ian Kelleher, 2025). And, these experiences contribute to disability and are an obstacle to productivity, particularly when onset occurs early in life (Gore et al., 2011). Attenuated forms are also reported in the general population among individuals without clinical impairment or clinical treatment needs, commonly referred to as psychotic-like experiences (PLEs) (I. Kelleher & Cannon, 2011). PLEs are relatively common, with lifetime prevalence estimated at 7–13% (Linscott & Van Os, 2013), and population surveys indicating that 11% of individuals report at least one psychosis-screening item (Scott, Chant, Andrews, & McGrath, 2006). Although many of these experiences are transient, longitudinal studies show that individuals with PLEs are at risk of later developing psychotic or other psychiatric disorders (Fisher et al., 2013; Rimvall et al., 2020). Taken together, these findings suggest that psychotic experiences are not confined to strict diagnostic categories, and vary along a continuum from subclinical expressions to clinical disorders. PLEs and clinical psychotic disorders also show overlapping demographic features and risk factors, consistent with the idea that they may share common etiological processes despite differing in clinical severity (Linscott & Van Os, 2013; McGrath et al., 2015; Van Os, Linscott, Myin-Germeys, Delespaul, & Krabbendam, 2009).

A recent fMRI twin study indicates that distinct dimensions of PLEs may have different etiological bases: distress shows moderate heritability, whereas frequency appears more strongly influenced by environmental factors (Şahin-Çevik et al., 2025). These findings highlight the importance of considering environmental exposures when examining mechanisms underlying PLE frequency. Environmental exposures such as traumatic life events (Beards et al., 2020; Cosgrave et al., 2021) and discrimination (Veling et al., 2007) have been shown to significantly influence progression along the psychosis spectrum, with risk increasing as these exposures accumulate (Cougnard et al., 2007). Composite indices such as the Maudsley Environmental Risk Score (Vassos et al., 2020) have been proposed to improve risk prediction (Mas et al., 2020).

Cognitive schemas that shape how individuals perceive themselves and others have been also reported in psychosis vulnerability. Increased negative self-evaluations have been associated with heightened psychotic symptomology (Tiernan, Tracey, & Shannon, 2014). In the non-clinical samples, individuals scoring high on paranoia have exhibited more negative self-schemas (Fowler et al., 2006; Humphrey, Bucci, Varese, Degnan, & Berry, 2021), and negative schemas have been shown to predict elevated levels of delusional thinking (Oliver, O’Connor, Jose, McLachlan, & Peters, 2012). Furthermore, individuals seeking help for psychosis and PLEs commonly exhibited negative beliefs about both themselves and others (e.g., ‘others are hostile’) (Taylor et al., 2014). These findings emphasize the importance of investigating PLEs in terms of both clarifying the developmental pathways of psychotic disorders and informing approaches to earlier recognition and preventive interventions in clinical settings.

From a neurobiological perspective, psychosis has been conceptualized as a disorder of disrupted brain connectivity (Friston, 1998). Alterations in network organization appear early in the illness course, and may precede clinical symptoms (Carletti et al., 2012). Structural and functional deviations in brain topology have also been reported in individuals with subclinical psychotic experiences (Drakesmith et al., 2015; Sheffield, Kandala, Burgess, Harms, & Barch, 2016). More recent work has identified widespread white matter changes in people with PLEs, including young women (Kjelkenes et al., 2025) and population-based samples (Schoorl et al., 2021), and atypical patterns of cortical gyrification have been associated with subclinical psychosis phenotypes (Evermann, Gaser, Besteher, Langbein, & Nenadić, 2020). Together, these findings suggest that early brain developmental markers may contribute to PLEs expression outside the clinical range. Network topology, in particular, has been proposed as a sensitive biomarker (Drakesmith et al., 2015), with deviations more pronounced in clinical than subclinical psychosis groups, but evident in both compared to healthy controls (Van Dellen et al., 2016). These insights provide a rationale for connectome-based approaches that assess whole-network integration, local-network segregation, communication between key nodes, and node clustering to capture structural signatures of psychosis risk.

Incorporating cumulative environmental risk, cognitive appraisals, and brain network organization in PLE samples offers a way to clarify mechanisms underlying the psychosis spectrum, while avoiding biases from treatment effects and illness-related complications. Although psychosis is recognized as heterogeneous, most studies have focused on single domains in isolation. Recent studies have applied machine learning approaches to identify neurobiological predictors of psychotic experiences (Kenney et al., 2022; Ma et al., 2023) and schizophrenia (Wu et al., 2022). However, integrative models that combine multiple modalities and validate their predictive value across independent cohorts remain limited. Identifying such predictors is important for understanding pathways from subclinical experiences to clinical disorder. In this study, we aimed to identify predictors that differentiate individuals with high versus low PLEs and validated these predictors in an independent external cohort consisting of first-episode psychosis patients, their unaffected siblings, and healthy controls. To achieve this, we applied a multimodal, data-driven framework incorporating environmental exposures, cognitive appraisals, clinical variables, cognitive functioning, and structural connectome measures. This approach allowed us to evaluate whether factors identified in a general population sample could reliably distinguish groups along the psychosis continuum. Through machine learning, we emphasized predictive accuracy while also examining the interpretability of contributing features. To our knowledge, this is the first study to jointly model environmental, cognitive, and neural factors in PLEs, and assess their relevance across both subclinical and clinical populations.

## Methods

### Participants

Participants were drawn from an in-house study on the genetic and environmental influences on brain development in healthy Turkish twins and siblings, recruited via community outreach and educational institutions in Turkey. In total, 417 individuals took part in the study: 363 twins, 6 triplets, and 48 siblings, with and without psychotic-like experiences (PLEs).

For the external validation cohort, three groups of participants were examined: first-episode psychosis (FEP) patients (*N* = 19), their siblings (*N* = 20), and healthy controls (*N* = 19). FEP patients were recruited from Ankara Bilkent City Hospital and diagnosed with the Structured Clinical Interview for DSM-5 Disorders: Clinician Version (SCID-5-CV). All were within 6 months of symptom onset and aged 14–23. Siblings were included if they were within 24 months of age of the proband.

Exclusion criteria across all groups included a history of neurological illness, intellectual disability, head injuries, or being a non-native Turkish speaker (based on self-report). For healthy participants only, a personal history of psychiatric illness was also an exclusion criterion. Additional exclusions were made for participants with missing T1-weighted (*N* = 5) and diffusion-weighted images data (*N* = 9), or failed structural connectivity matrix reconstruction, or more than 10 regions with a rank of zero (*N* = 5). In the PLE cohort, two individuals were excluded due to missing CAPE-42 scores. Demographic information is presented in Table 1.

**Table 1.** Demographic characteristics of the sample

Psychotic-like experiences sample (*N* = 396)			
Sample characteristics		Minimum	Maximum
Age, mean (SD)	19.72 (2.5)	14	24
Sex			
Females, *n* (%)	249 (62.9)		
Males, *n* (%)	147 (37.1)		
Zygosity			
Monozygotic, *n* (%)	141 (35.61)		
Dizygotic, *n* (%)	211 (53.28)		
Triplets, *n* (%)	6 (1.52)		
Sibling, *n* (%)	38 (9.6)		
IQ, mean (SD)^a^	102.10 (13.50)	70	154
Psychotic-like experiences group, *n* (%)^b^			
High psychotic-like experiences	196 (49.5)		
Low psychotic-like experiences	200 (50.5)		

External validation cohort (*N* = 58)			
	Patients (*n* = 19)	Minimum	Maximum
Age, mean (SD)	20 (3.16)	15	25
Sex			
Females, *n* (%)	7 (36.8)		
Males, *n* (%)	12 (63.2)		
IQ, mean (SD)^a^	79.33 (17.44)	56	108

	Siblings (*n* = 20)	Minimum	Maximum
Age, mean (SD)	18.40 (3.76)	13	25
Sex			
Females, *n* (%)	10 (50)		
Males, *n* (%)	10 (50)		
IQ, mean (SD)^a^	90.40 (14.29)	63	128

	Healthy controls (*n* = 19)	Minimum	Maximum
Age, mean (SD)	19.11 (2.40)	14	24
Sex			
Females, *n* (%)	7 (36.8)		
Males, *n* (%)	12 (63.2)		
IQ, mean (SD)^a^	104.74 (15.35)	70	135

Abbreviation: SD, standard deviation.

a Block Design and Matrix Reasoning subtests of the Wechsler Abbreviated Scale of Intelligence, Second Edition (WASI-II) was used to obtain IQ scores.

b Participants were divided into high and low PLE groups based on the median score of CAPE-42 total score. Scores above the median were placed in the high PLEs group, while scores at or below the median were placed in the low PLE group.

The study was approved by the ethics committees of Ankara University Medical Faculty and Bilkent University. All participants provided written informed consent; for those under 18, consent was provided by a parent or legal guardian.

### Clinical and subclinical measures

#### The community assessment of psychic experiences-42 (CAPE-42)

Psychotic-like experiences (PLEs) were assessed using the CAPE-42, a self-report questionnaire assessing the frequency and distress associated with PLEs in the general population (Konings, Bak, Hanssen, Van Os, & Krabbendam, 2006; Mark & Toulopoulou, 2016; Stefanis et al., 2002). It consists of 42 items that evaluate positive, negative, and depressive dimensions of experience.

We utilized the total CAPE-42 frequency score, which was obtained by summing the frequency ratings across all the items. Participants were then dichotomized into low and high PLEs groups based on the median of the total frequency score. Scores above the median were placed in the high PLEs group, while scores at or below the median were placed in the low PLEs group.

### Environmental and protective measures

The environmental assessments included paternal age, obstetric and delivery complications, winter birth in the Northern Hemisphere, ethnicity, urbanicity, parental social class, lifetime cannabis usage, tobacco usage, alcohol consumption, childhood trauma, discrimination, hearing impairment, threatening life events, informal social control, participants’ relationship status, religion status, and social cohesion and trust (Section 1 in Supplementary). We also included the Maudsley Environmental Risk Score (ERS), a cumulative index of six validated environmental risk factors: ethnic minority status, urbanicity, paternal age, obstetric complications, cannabis use, and childhood adversity (Vassos et al., 2020). When individual variables were part of the composite ERS (e.g., childhood trauma), only the variable which has a higher odds ratio from generalized estimating equations was included to avoid redundancy and ensure a parsimonious predictor set for the model.

### Cognitive and cognitive self-schema measures

#### Estimated general function and fluency

The quick measure of Block Design and Matrix Reasoning subtests of the revised Wechsler Abbreviated Intelligence Scale, Second Edition (WASI-II), was used as a proxy of general intelligence (Wechsler, 2011). The Perceptual Reasoning Index composite score was used to measure non-verbal and fluid reasoning. Verbal fluency was assessed through the verbal fluency task (Section 2 in Supplementary).

#### Brief core schema scales (BCSS)

BCSS is a 24-item self-report questionnaire and was used to assess the participants’ own schemata for one’s self and others (Fowler et al., 2006). Appraisals of self include negative-self (6 items, e.g., ‘I am worthless’) and positive-self (6 items, e.g., ‘I am valuable’), while appraisals of others include negative others (6 items, e.g., ‘Others are hostile’) and positive others (6 items, e.g., ‘Others are truthful’). The items were rated dichotomously (Yes/No), followed by a degree of belief conviction consisting of a Likert scale from 0 to 4 (‘believe it slightly’ to ‘believe it totally’) for endorsed items. Scores for each domain were calculated by summing the items.

#### Family history of psychiatric disorders

Family Interview for Genetic Studies (FIGS) is a self-report questionnaire (Maxwell, 1992). It was used to assess the presence or absence of psychiatric disorders among participants’ first-degree relatives.

### Neuroimaging

#### Structural connectome construction

Detailed information is provided in Section 3 in Supplementary. Briefly, for each individual, a structural brain connectome was reconstructed using diffusion-weighted and T1-weighted MRI data. Cortical parcellation was performed on T1-weighted images using FreeSurfer (FreeSurfer 7.1.0, http://surfer.nmr.mgh.harvard.edu/). Diffusion-weighted images were preprocessed with the FSL tool (FSL 6.0, https://fsl.fmrib.ox.ac.uk/fsl/fslwiki) to correct for susceptibility distortions, eddy currents, and motion artifacts (Jenkinson, Beckmann, Behrens, Woolrich, & Smith, 2012). Connectivity Analysis Toolbox (CATO v2.5, http://www.dutchconnectomelab.nl/CATO) was integral in integrating these steps and facilitating the creation of a 114 x 114 connectivity matrix, where nodes represented brain regions, while edges quantified white matter fiber tracts (de Lange, Helwegen, & van den Heuvel, 2023). Next, the Brain Connectivity Toolbox in MATLAB R2020A (http://www.brain-connectivity-toolbox.net) was used to compute key graph theoretical metrics, including global efficiency, density, reflecting whole-brain network integration; local efficiency, betweenness centrality, and clustering coefficient, highlighting localized structural network characteristics (Rubinov & Sporns, 2010) (Supplementary Table S1).

### Statistical analyses

In the main dataset, the outcome is a binary variable based on the median CAPE-42 frequency score (≤73 = low, > 73 = high). This enables comparability with the categorical structure of the external validation cohorts (patients, siblings, and controls) and aligns with previous epidemiological and machine learning studies utilizing binary outcomes (Dominguez, Wichers, Lieb, Wittchen, & Van Os, 2011; Haroon et al., 2018; Krämer et al., 2023; Tandon et al., 2012).

In the external validation, the classification outcome was defined according to clinical and familial status, which was defined as patient–sibling or patient–healthy. For the evaluation of the developed model on external sets, the predicted positive class (coded as 1) comprised patients with first-episode psychosis (FEP), while the predicted negative class (coded as 0) comprised their unaffected siblings in the first external dataset, and demographically matched controls (HC) in the second.

For zygosity, monozygotic twins and triplets were retained as separate groups, while dizygotic twins, and non-twin siblings were combined, given their comparable degree of genetic relatedness.

Group differences in PLEs across these categories were tested with Chi-square while differences in age and sex between study groups were assessed using appropriate statistical tests (Section 4 in Supplementary).

All demographic analyses were done using IBM SPSS Statistics (Version 25), using a significant threshold of *p* < 0.05.

To address the issue of having more variables (*N* = 741) than observations (*N* = 396), we conducted univariate Generalized Estimating Equation (GEE) models to identify significant associations with PLEs, clustering families within pairs to account for family effects. Our focus was not on modeling these effects. This approach mitigated overfitting and multicollinearity, allowing for a more manageable set of variables for the machine-learning phase. In the GEE analysis, we used an exchangeable correlation structure, as there was no logical order among sibling pairs and the data did not follow a time series. GEE results were reported as odds ratios with a 95% confidence interval, with a significance level set at 0.05. Following this, a correlation filter was applied to eliminate highly collinear variables for the elastic model.

#### Machine learning

In this study, we simultaneously examined the relationship between variables using machine learning (ML) methods. Elastic net modeling was used since it was designed to enhance the performance of the classic regression methods when there are many predictor variables (Rosenström et al., 2018). It reduces multicollinearity and overfitting of the model through regularization (Zou & Hastie, 2005). The following preprocessing procedures were performed to prepare the data prior to ML. First, dummy variable coding was performed for categorical variables with more than two categories. Second, participants with missing values among the selected variables were excluded to consider complete cases. Third, the dataset was randomly divided into a training set comprising 70% of the data and a test set comprising the remaining 30% using a hold-out approach. This method involves randomly splitting the data into training and testing sets. The model was developed during the training phase and evaluated using an unseen test set, with particular attention given to preserving the prevalence in both sets during the random split. Lastly, z-standardization was applied to the training set to obtain standardized coefficients for the ML models and ensure comparability across variables. Based on the characteristics of the training set, the test set was also standardized to ensure consistency and prevent data leakage. Following the preprocessing steps, the Least Absolute Shrinkage and Selection Operator (LASSO) model was used on the train set. The primary purpose of LASSO regression is variable selection, achieved by setting the weights of unimportant variables to zero. Variables with non-zero coefficients were retained for the model (Tibshirani, 2011). Subsequently, to classify two classes of PLEs by using the most predictive variables identified in the training set, a classification model was developed using the Elastic Net algorithm, which combines LASSO and Ridge methods to optimize variable weights rather than solely focus on selection. Thus, LASSO identified important variables, while the Elastic Net established the final classification model. Technical implementation details are reported in Section 5 in Supplementary.

Because of the data-driven approach of the study, no hypothesis test was conducted regarding model performances; however, 95% confidence intervals were provided along with the model’s AUC values; intervals excluding 0.50 were interpreted as evidence of meaningful classification.

## Results

### Psychotic-like experiences sample

#### Statistical analysis

PLE distributions did not differ across zygosity (X*^2^*, *p* = .99), and no significant group differences were found for age (Mann–Whitney U, *p* = .85) or sex (X^2^, *p* = .43; see Supplementary Tables S2–S3).

GEE models were first applied to identify variables significantly associated with PLEs. Out of 741, the analysis identified 37 significant predictors (*p* < 0.05). Insignificant age, sex, and PRI composite score variables were kept as covariates, resulting in 40 explanatory variables in this step. The ERS emerged as a stronger predictor than physical neglect, emotional neglect, and total score for childhood trauma variables, leading to the exclusion of these childhood trauma-specific measures in favor of ERS in further analyses (Supplementary Table S4).

Spearman correlations revealed that several eloc and cc regions were highly correlated (rho >0.70; Supplementary Table S5). We excluded highly correlated cc, yielding 34 explanatory variables for further analysis.

#### Machine learning

**Figure 1.**
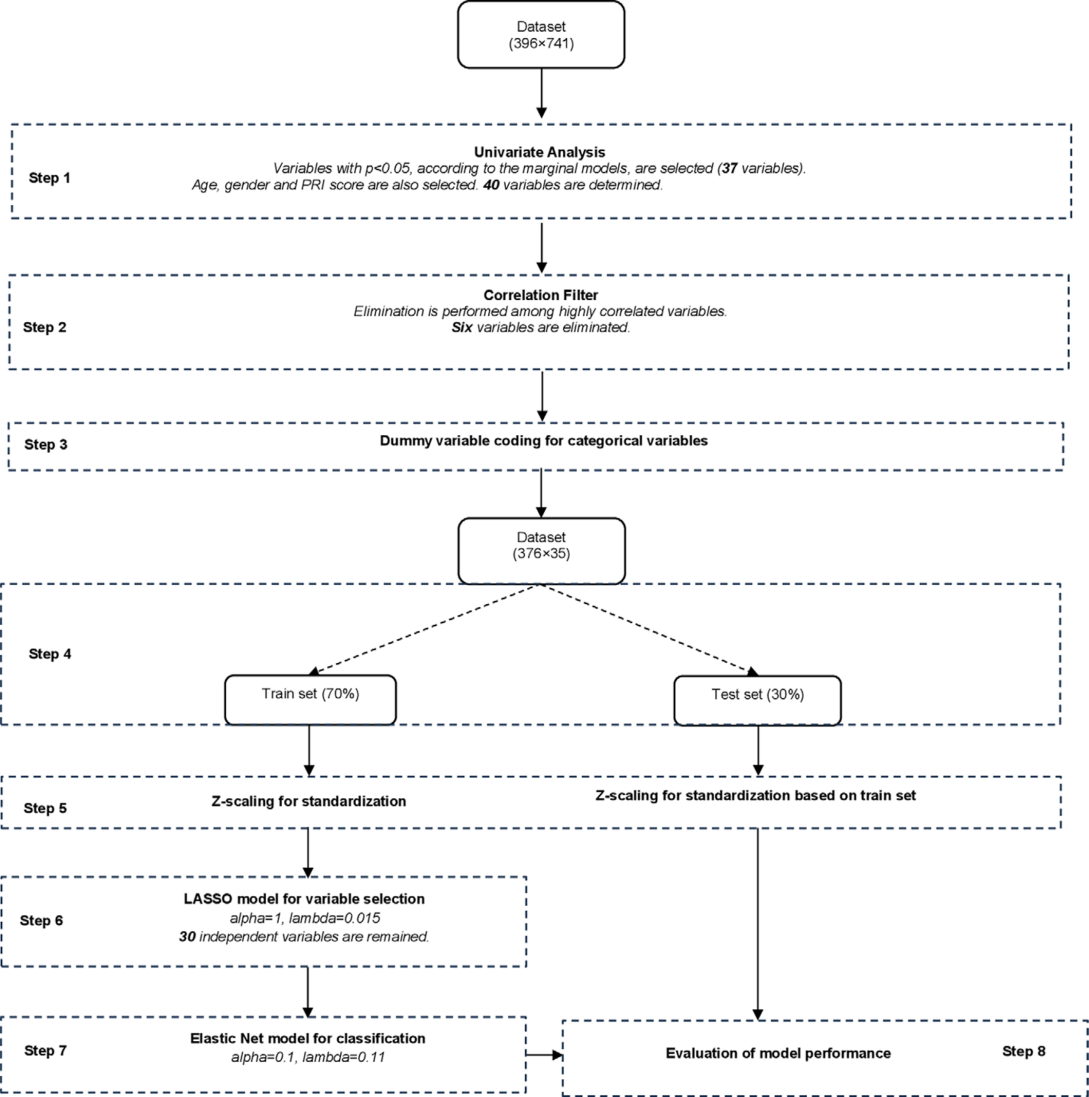
Overview of data processing and model development steps. This flowchart shows the full pipeline used in the study, starting with the initial dataset and progression through univariate filtering, correlation-based variable elimination, and dummy coding for categorical data. The dataset was split into a training set (70%) and a test set (30%), with z-standardization applied using training set characteristics. Feature selection was performed using the LASSO model (α = 1, λ = 0.015), and classification was completed using the Elastic Net algorithm (α = 0.1, λ = 0.11). The final model’s performance was evaluated on the test set. Abbreviation: PRI = perceptual reasoning index.

LASSO regression was employed to conduct a multivariate evaluation, identifying the most important variables selected during the initial GEE analysis. Of the 396 participants retained for the GEE analysis, 20 were excluded in this step because of missing data on selected predictors, resulting in 376 complete cases. Furthermore, dummy coding was applied to parental social class, the only categorical variable with more than two levels among the selected predictors. Thirty-five z-standardized variables were finally included in this step. A detailed explanation is provided in Section 6.3 in Supplementary. A visual summary of the analysis steps is provided in Figure 1.

Variable selection using LASSO was conducted on the training set as explained above. Following this, LASSO identified 27 key explanatory variables (Section 6.4 in Supplementary).

**Figure 2.**
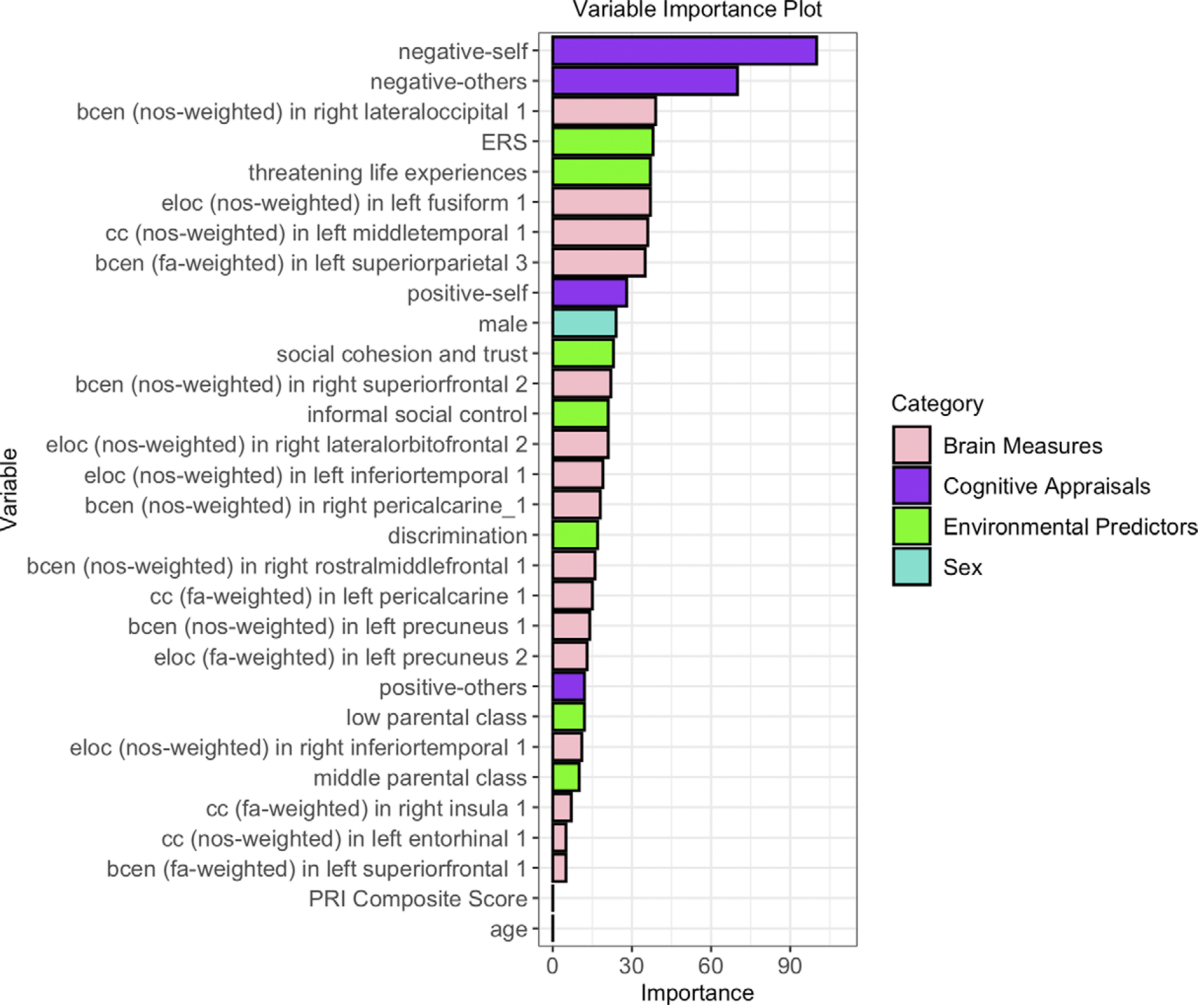
Variable importance plot for predicting psychotic-like experiences. The plot displays all the selected variables ordered based on their standardized coefficients, indicating their variable loading to predict psychotic-like experiences. *Note:* Variables are colored based on their categories: environmental predictors (green), cognitive appraisals (purple), brain measures (pink), and sex (blue). Abbreviations: bcen, betweenness centrality; cc, clustering coefficient; eloc, local efficiency; ERS, environmental risk score for psychosis; FA, fractional anisotropy; NOS, number of streamlines; PRI, perceptual reasoning index.

The variable importance plot (Figure 2) reflects the standardized coefficients for predictors contributing to the classification of PLEs, ranked in descending order, while SHAP (SHapley Additive exPlanations) explainer method illustrates how each predictor influences the low and high PLE classes (see Section 6.5 and Figure S1 in Supplementary).

Negative-self and negative-others schemas emerged as the strongest contributors. Among environmental features, ERS showed the highest contribution, followed by threatening life experiences, social cohesion and trust, informal social control, discrimination, and parental social class (low and middle) variables. Positive-self and positive-others variables were also retained, albeit with lower weightings.

Among the structural network topology measures, betweenness centrality, local efficiency, and clustering coefficient, representing nodal network properties, were important PLEs’ predictors. FA-weighted network properties showed a clear asymmetry between the hemispheres, with a stronger predictive contribution from the left hemisphere (five regions), compared to the right hemisphere (one region).

#### Evaluation of the model performance

As shown in Table 2, the model had an accuracy of 75.2%, correctly identifying 36 out of 55 high-PLE individuals (sensitivity = 0.655) and 49 out of 58 low-PLE individuals (specificity = 0.845). The positive predictive value was 0.80, highlighting the model’s moderate ability to correctly identify individuals with high PLEs, while the negative predictive value was 0.721, indicating the model had reasonable classification of low-PLE cases (72.1%). The model’s overall discriminatory power (AUC = 0.750) was moderate in distinguishing between high and low PLEs.

**Table 2.** Confusion matrix and performance metrics of high–low psychotic-like experiences

Metric				
Confusion matrix^a^		Class^b^		
	Predicted class	High	Low	Total
	High	36 (TP)	9 (FP)	45
	Low	19 (FN)	49 (TN)	68
	Total	55	58	113

Performance metrics	Value			
Accuracy	0.752			
Sensitivity	0.655			
Specificity	0.845			
Positive predictive value	0.800			
Negative predictive value	0.721			
F1	0.720			
AUC (95% C.I.)	0.750 (0.671–0.829)			

Abbreviations: AUC, area under the receiver operating characteristic curve; CI, confidence interval.

a The confusion matrix summarizes the number of true positives (TP), true negatives (TN), false positives (FP), and false negatives (FN) to perform a clear evaluation of model performance.

b Low class included participants with CAPE-42 frequency scores equal or lower than the median (low PLEs) while high class included those with scores above the median (high PLEs).

### External validation cohort

First-episode of psychosis (FEP), their siblings, and healthy control (HC) groups were used to assess whether the important predictors identified for PLEs could effectively distinguish these groups. After excluding individuals with missing values on key predictors or covariates, the final validation sample in this step included 16 FEP patients, 19 siblings, and 18 HCs. The Shapiro–Wilk tests confirmed normality of age distribution among FEP and HC groups (W = 0.95, *p* = 0.15). The groups did not differ in age (t (26) = 0.127, *p* = 0.90) and sex (X^2^(1) = 0.83, *p* = 0.77). Descriptive statistics are provided in Supplementary Tables S7 and S8.

#### Patient–sibling (genetic high risk) dataset

The performance results for distinguishing the FEP patients from their healthy siblings are presented in Table 3.

**Table 3.** Confusion matrix and performance metrics of patient–sibling

Metric				
Confusion matrix		Class^a^		
	Predicted class	Patient	Sibling	Total
	Patient	7 (TP)	3 (FP)	10
	Sibling	9 (FN)	16 (TN)	25
	Total	16	19	35

Performance metrics	Value			
Accuracy	0.657			
Sensitivity	0.434			
Specificity	0.842			
Positive predictive value	0.700			
Negative predictive value	0.640			
F1	0.539			
AUC (95% C.I.)	0.640 (0.489–0.791)			

Abbreviations: AUC, area under the receiver operating characteristic curve; CI, confidence interval; FN, false negative; FP, false positive; TN, true negative; TP, true positive.

a Patient class included FEP patients while siblings class included healthy siblings.

The model correctly classified 7 instances out of 16 patients and 16 instances out of 19 siblings. It misclassified 3 siblings as patients and 9 patients as siblings.

The overall accuracy was 65.7%, with a sensitivity of 0.434 and a specificity of 0.842. The positive predictive value was 0.70, while the negative predictive value was 0.64. The AUC value of 0.640 reflected the model’s modest discriminative ability, particularly with relatively better performance in identifying siblings.

#### Patient–healthy controls dataset

The model had a weak performance with a poor accuracy of 0.412 and AUC levels of 0.590 (95% CI: 0.420–0.760) in distinguishing between FEP patients and healthy controls (Supplementary Table S9).

## Discussion

The primary goal of this study was to clarify the multimodal mechanism underlying psychotic-like experiences (PLEs) by integrating environmental exposures, cognitive appraisals, clinical measures, cognitive functioning, and structural connectome topology. Through a structured analytical pipeline- combining univariate screening, correlation filtering, and LASSO for feature selection- we reduced 741 candidate variables to 30 predictors, which were then applied to the Elastic Net for classification.

A secondary aim was to test the generalizability of these predictors in an external cohort of first episode of psychosis patients, their siblings, and healthy controls. Although trained on a non-clinical sample, the model demonstrated discriminative ability, albeit modest in identifying patients and relatively stronger performance in distinguishing siblings from patients. Including an independent clinical cohort enabled us to explore whether the model’s findings generalize beyond non-clinical samples. The external validation supports the potential for translating insights from subclinical PLEs to clinically relevant distinctions.

Our findings suggest that PLEs may reflect the convergence of maladaptive cognitive appraisals, cumulative environmental burdens, and disrupted brain network architecture. Among environmental predictors, the environmental risk score emerged as the strongest predictor – surpassing individual environmental variables – by capturing the aggregate effect of multiple environmental adversities. This supports the view that psychosis risk is associated more with cumulative environmental load (Cougnard et al., 2007; Stepniak et al., 2014) and highlighting the need to study these exposures collectively rather than as isolated predictors (Vassos et al., 2020).

Negative-self and negative-others were the strongest predictors of PLEs, consistent with prior work linking maladaptive self-perceptions and interpersonal evaluations to psychotic symptoms (Jaya, Ascone, & Lincoln, 2017; Kesting & Lincoln, 2013; Taylor et al., 2014). Similarly, a casual discovery analysis in the same but smaller cohort showed that negative self-schemas exert a direct influence on psychosis-proneness, suggesting that such cognitive structures may act as vulnerability markers by shaping the impact of environmental stressors (Sahin-Ilikoglu et al., 2025).

While threatening life events (e.g., a serious injury), discrimination, and informal social control (e.g., collective intervention to discourage deviant behaviors) were risk factors, social cohesion and trust, and positive schemas acted as protective factors. These findings suggest that social contexts may not be merely a backdrop for risk but play an important role in modulating whether subclinical risk remains benign or progresses toward clinical expression.

Our findings revealed a distinct pattern of brain network alterations associated with PLEs, particularly in betweenness centrality. Increased centrality was observed in some cortical hubs like the superior frontal gyrus, precuneus, and reduced centrality in pericalcarine cortex – contrasting with schizophrenia, where widespread reductions in centrality are typical (Van Den Heuvel, Mandl, Stam, Kahn, & Hulshoff Pol, 2010; Zhang et al., 2012). Our findings may reflect early-stage reorganization, while some hubs become more central, others are downregulated. Such shifts may reflect compensatory adaptations or transitional stages (Drakesmith et al., 2015) in the network topology that emerge before the more generalized connectivity disruptions seen in clinical psychosis.

The left-lateralized network contributions to PLEs align with findings of disrupted hemispheric asymmetry in schizophrenia (Ribolsi, Daskalakis, Siracusano, & Koch, 2014; Ribolsi et al., 2009), suggesting early white matter hemispheric imbalances as a neurodevelopmental vulnerability.

The model’s ability to distinguish individuals with high versus low PLEs reflects the value of integrating cognitive appraisals, environmental exposures, and structural brain connectome features. With an AUC of 0.75 and a specificity of 84.5%, the model showed a moderate ability to identify low-risk individuals based on multidimensional risk profiles. It also indicates that the well-established psychosis-related factors in the literature are also crucial for PLEs, reinforcing the view that PLEs lie on a continuum with psychotic disorders.

The model demonstrated a modest discriminative ability in distinguishing unaffected siblings from FEP patients, with high specificity (0.842) but lower sensitivity (0.434). This asymmetry may suggest that some of the predictors, while not always aligning with overt clinical presentations, are more consistently present in siblings. These patterns may indicate a broader vulnerability to psychopathology across the continuum rather than pinpointing a clinical state. Additionally, their presence in unaffected siblings may reflect underlying resilience traits. Thus, the same variables predicting PLEs might serve dual roles: highlighting shared susceptibility across at-risk individuals while also capturing protective mechanisms that enable some to remain unaffected despite genetic or environmental vulnerabilities. Therefore, siblings may have intact cognitive appraisal patterns, social buffering, or preserved network structures that decrease the symptom emergence and support mental health stability. Supporting this, a recent study using classical twin modeling within the same cohort has indicated that PLE frequency shows environmental influences, whereas distress reflects moderate heritability (Şahin-Çevik et al., 2025). Although genetic influence cannot be ruled out, our findings emphasize the weight of environmental exposures and protective factors in PLE frequency. Overall, we suggest that PLE-based models could help identify both risk markers and resilience factors, which may be valuable for early detection and preventive strategies. Further, studies with larger samples, such as the Adolescent Brain Cognitive Development (ABCD) study, could confirm this observation (Karcher & Barch, 2021).

Several limitations warrant consideration. First, we dichotomized total CAPE-42 frequency scores at the median in the main dataset. Although this helped compare low and high PLEs, dichotomizing continuous variables may lower statistical power and oversimplify symptom severity variation, potentially affecting the strength or accuracy of observed associations.

Second, recruiting participants such as twins, triplets, and siblings may limit generalizability, and even though twin pairs were treated as clusters to account for their similarity, it could have contributed to differences in model performance when applied to more genetically diverse cohorts, such as FEP patients, their siblings, and healthy controls as it was done here. Third, adding polygenic risk score (PRS) for schizophrenia into our models, or selecting participants with PLEs and high PRS could have led to different outcomes. Fourth, as the study aimed to develop predictive models rather than variance decomposition, classical twin modeling was not applied. Future work could adopt twin modeling to better disentangle genetic and environmental contributions to PLEs.

Fifth, some variables – including substance use, trauma exposure, and family psychiatric history – relied on self-reported data, making them susceptible to recall bias, underreporting, and subjective interpretation.

Sixth, our general intelligence measure did not incorporate a verbal subtest, and it may not fully approximate general intelligence as in dyad-based approaches that combine verbal and performance domains (Girard, Axelrod, Patel, & Crawford, 2015).

Seventh, model performance was the weakest in distinguishing patients from healthy controls. One possible explanation is that predictors derived from PLEs in non-clinical samples may be more sensitive to variation within healthy populations than to differences that define patient-control status, thus making it more challenging for the model to detect clear boundaries between these groups. Additionally, our dataset lacked state-dependent clinical features such as current symptom severity or medication use, which may have further influenced the model performance. Nevertheless, the model demonstrated high specificity when distinguishing patients from their unmedicated siblings, which suggests that the discriminative patterns it captured may extend beyond medication effects and could reflect more stable, familial or trait-level markers. Eighth, the unexpected protective effect of low parental class is challenging to interpret, but may reflect resilience processes such as shift-and-persist (Chen, Lin, Zhao, & Chi, 2025). Ninth, while our study emphasizes the significance of negative-self, it does not clarify cognitive-emotional processes underlying these associations. Further research should address these gaps. Tenth, the small sample sizes in external validation cohort may have further limited statistical power (Figueroa, Zeng-Treitler, Kandula, & Ngo, 2012; Jo et al., 2020).

Finally, model performance was evaluated using a single train-test split, which may be sensitive to data partitioning. Nonetheless, the inclusion of an independent external validation cohort strengthens the generalizability of our findings. Future studies could enhance robustness through repeated cross-validation or bootstrapping.

## Conclusion

The current study demonstrates that PLEs are associated with both resilience and vulnerability markers across cognitive, environmental, and neurobiological domains. Considering these factors together provides a better perspective on the mechanism underlying variation along the psychosis spectrum. Predictors identified in a non-clinical sample partially generalized to clinical cohorts can suggest that PLE-based models capture not only markers of risk but also indicators of resilience that help explain why some individuals remain unaffected despite familial vulnerability. Clinically, these findings support preventive approaches to strengthen resilience and address modifiable vulnerabilities in at-risk populations.

## Supporting information

Arslan et al. supplementary material Arslan et al. supplementary material

## Supplementary material

The supplementary material for this article can be found at http://doi.org/10.1017/S0033291725102201.
