# Supplementary material for: Multimodal prediction of psychotic-like experiences using elastic net modeling: external validation in a clinical sample
Source: Psychol Med. 2025 Nov 14;55:e346. doi: 10.1017/S0033291725102201 (PMC13058658; doi:10.1017/S0033291725102201)
Supplement: Arslan et al. supplementary material [file S0033291725102201sup001.pdf]

Supplementary Materials

1. Environmental and Protective Measures

The amended version of the Medical Research Council (MRC) Sociodemographic Form,(Mallet, 1997) part 1 was used to collect information on ethnicity, urbanicity, parental social class, paternal age, and hearing impairment. **Ethnicity** was assessed by asking the participant's ethnic identity and **urbanicity** by taking into account the "birthplace" of the participant. The classification of the urban/rural level was examined through the open source "European Commission Global Human Settlement (GHS)" (https://ghsl.jrc.ec.europa.eu/ESMVisualisation.php). **Parental social class** was assessed by taking into account the occupation of the participant's mother or father, who had a higher occupational level. The form included ten classes: higher grade professional, lower grade professional, intermediate occupations, small employer, self-employed occupations, lower supervisory and lower technician occupations, lower technical occupations, routine occupations, never worked, and long-term unemployed. **Paternal age** was assessed by asking about the father's age at the participant's birth, while **hearing impairment** was assessed by asking about the absence/presence of lifetime hearing impairment. The amended version of MRC Sociodemographic Form part 2 was used to assess the **participant's relationship status** by asking whether the participant is single, married/living with someone, or in a steady relationship at the time of the assessment, and **religion** by asking the participant's religious affiliation. **Winter birth** consisted of information on whether the participant was born in the winter months in Northern hemisphere (December, January, February). **Obstetric complications** were assessed

by asking participant's mother whether she experienced any of the complications in the subcategories at the participant's birth. Subcategories included preeclampsia, preterm placenta ablation, blood group intolerance, rubella or syphilis, bleeding, feverish infections, preterm rupture, preterm contractions, preterm birth, breech birth, imminent asphyxia, birth traumas (Preti et al., 2012). **Cannabis usage** was assessed as lifetime cannabis use frequency by using the Cannabis Experience Questionnaire and categorized into three groups, which are "no use", "little or few times a year," and "monthly or above" (Di Forti et al., 2009). **Tobacco usage** included information on the participants' number of cigarettes smoked per day within 12 months. **Alcohol consumption** included information on the number of units of alcohol consumed by the participant in 1 week and one year. The number of units in the alcohol was calculated by multiplying the total volume of alcohol contained in the drink (ml) by its alcohol strength (ABV, i.e., alcohol by volume) and then dividing the result by 1.000. The formula for the calculation was adapted from "National Health Service United Kingdom (NHS-UK)" (https://www.nhs.uk/live-well/alcohol-advice/calculating-alcohol-units/). Both tobacco and alcohol consumption were assessed through The European Network of National Schizophrenia Networks Studying Gene-Environment Interactions (EU-GEI) Tobacco and Alcohol Questionnaire (Sánchez-Gutiérrez et al., 2023). **Discrimination** was assessed by the number of lifetime unfair experiences in twelve domains, such as education and general medical care. Presence/absence of the discrimination, number of times the unfair experience, participant's age at first occurrence, and participant's attribution for the underlying reason for the unfair experience (e.g., race, age, mental illness) were included in the questionnaire for each domain (Gabbidon et al., 2014). Only the number of the unfair experiences were employed in this study. **Threatening life events** were assessed using the EU-GEI List of Threatening Events

questionnaire, which consisted of 20 items. Participants were asked about the presence/absence of stressful events (e.g., a serious injury or separation from a partner) that happened twelve months prior to the study (Brugha & Cragg, 1990). **Social cohesion and trust** were assessed through the EU-GEI Social Environment Assessment Tool, including civic disorder, the impact of civic disorder, informal social control, social cohesion and trust, ethnic diversity, physical disorder, and individual community participation (Kirkbride et al., 2008). Social cohesion and trust were used in this study to enquire about the quality and quantity of social interactions between the neighbors. **Informal social control** was used to evaluate the eagerness of the residents in the same neighborhood to interfere with the neighborhood problems. Higher scores in subdomains of informal social control, social cohesion, and trust suggest a more positive sense of the residents for the local community. **Childhood trauma** was assessed through Childhood Trauma Questionnaire, short version (CTQ) consisting of experiences of emotional abuse (5 items, e.g., "People in my family said hurtful or insulting things to me") and emotional neglect (5 items, e.g., "I felt that someone in my family hated me"), physical abuse, (5 items, e.g., "My family members were hitting me so violently that I had bruises or scratches on my body"), physical neglect (5 items, e.g., "I had to wear dirty clothes"), and sexual abuse (5 items, e.g., "I believe that I was sexually abused") before the age of 17. It is a retrospective self-administered questionnaire that includes 25 items that ask participants about the frequency of the experiences, varying from "never" to "very often" (Bernstein et al., 2003).

1.2. Environmental Risk Score for Psychosis (ERS)

A single environmental risk score was calculated for each participant to evaluate the measure of environmental risk factors for psychosis in aggregate (Vassos et al., 2020). For the calculation, the following variables and scoring were used. Urbanicity was scored based on whether the participant was born in a rural, town area/suburb, or urban center. Each birthplace was given points of -1.5, 0, and 1, respectively. Cannabis was scored based on the lifetime frequency of using cannabis in which included no exposure, little or a few times a year or a few times a month & above. Each frequency was given points of -1, 0, and 3, respectively. Childhood trauma was scored based on the absence/presence of psychological, physical abuse and neglect, sexual abuse, bullying, and parental death before the age of 17. In the case of absence of trauma, -1.5 points were given, while 2.5 points were given in any exposure to childhood trauma. Ethnicity was scored based on whether the participant was a Turkish native or a migrant. 0 or 2 points were given, respectively. Paternal age was scored based on the father's age at the participant's birth. Paternal age for less than 40 years old, 0 points; for between 40-50 years old, 0.5 points; for older than 50 years old, 2 points were given. The obstetric complications were scored based on the difficulty during the mothers' pregnancy or delivery of the participants. While the absence of complications received -0.5 points, severe complications such as preterm rupture received 1.5 points. A single environmental risk score was obtained by summing the scores in each category.

1.3. Categorization and Scorings of the Variables

Before entering the variables into the model, we categorized the variables as follows: for the ethnicity, Turkish citizens were coded as 1 while the other ethnicities were 2. Parental social class was divided into three categories, which are low, middle, and high. Low parental social class was coded as 1 and included "never worked and long-term unemployed, lower supervisory and lower technician occupations, lower services, sales, and clerical occupations, lower technical occupations, routine occupations"; the middle parental class was coded as 2 and included "intermediate occupations, small employer and self-employed occupations, self-employed occupations" while high parental social class was coded as 3 and included "higher grade Professional, lower grade professional." Urbanicity was divided into two categories, which included no urban birth (i.e., dense and semi-dense urban cluster, suburban cells, rural cluster, low density; coded as 0) and urban birth (i.e., urban center, coded as 1). Hearing impairment was divided into the absence of hearing impairment (coded as 0) and the presence of hearing impairment (coded as 1). Participants' relationship statuses were divided into two categories: single (coded as 1) and in relationship (coded as 2). Religion was also divided into two categories: non-believer (coded as 0) and believer (coded as 1). Winter birth was also divided into two categories, which included the absence of winter birth (coded as 0) and the presence of winter birth (coded as 1). Obstetric complications were examined as two separate variables, which are obstetric complications during pregnancy and obstetric complications during birth. Both variables were divided into two categories: the presence of complications (coded as 1) and the absence of complications (coded as 2). Cannabis frequency was divided into three categories: no usage (coded as 0), little or few times a year (coded as 1), and monthly or above (coded as 2).

Medication usage was divided into two categories: no psychiatric medication usage (coded as 0) and presence of psychiatric medication (coded as 1). Family history of psychiatric disorder was divided into two categories, which are the absence of the psychiatric disorder (coded as 0) and the presence of psychiatric disorders, including psychosis, obsessive-compulsive disorder, autism, depression, or mania, in the first-degree family member (coded as 1). To calculate the total score for discrimination, the number of unfair experiences was added up. The score for threatening life events was calculated by adding the yes answers. For both informal social control and social cohesion and trust score calculation, all the item answers were coded from 1 to 5, and then, item scores were summed up for each subdomain. To calculate the CTQ scores, first, all the items in emotional abuse, physical abuse, and sexual abuse were coded from 1 to 5. All items in the emotional neglect and two items in the physical neglect were reverse coded. After that, item scores were summed up for each subdomain.

2. *Estimated general function and fluency*

2.1 Wechsler Abbreviated Scale of Intelligence, Second Edition (WASI-II)

Block design includes 13 items and reflects the analysis and synthesis ability for the abstract visual stimuli, while Matrix Reasoning includes 30 items and reflects the fluid and broad visual intelligence, spatial capacity, and classification ability in the knowledge of simultaneous processing and the perceptual organization of the participants. The raw scores of the examinee in subtests were converted into age-dependent scale scores. Corresponding T scores of each subtest

were calculated before summing them up. Then, the sum of T scores was converted into Perceptual Reasoning Index (PRI) (Wechsler, 2011).

2.2 Verbal Fluency

Verbal fluency task consisting of semantic and phonemic fluency (Benton, 1968; Newcombe, 1969) was used to evaluate verbal ability and executive control (Shao et al., 2014). Participants were instructed to generate as many unique words as possible within one minute per category (Borkowski et al., 1967). To measure semantic fluency, participants were asked to generate words related to categories of animals and fruits, respectively. For phenomic fluency, they were requested to generate words beginning with the letters A and S, respectively. Then, the numbers of the valid words in each category were counted to reflect semantic and phenomic fluency scores.

3. Neuroimaging

3.1. MRI Data Acquisition

To reconstruct the structural brain connectivity circuits, diffusion-weighted and T1-weighted magnetic resonance imaging (MRI) were utilized. MRI data acqusition was performed at Aysel Sabuncu Brain Research Center, Bilkent University.

Magnetic resonance imaging was performed with a 3 Tesla MRI system using a head coil (Siemens Magnetom). Diffusion-weighted images of the brain were recorded in a straight axial plane by applying 30 gradient directions with a single shot-EPI (Echo Planar Imaging) sequence. Duration of imaging sequence = 7 minutes 22 seconds, TE = 102 ms, TR = 10740 ms, voxel size = 2 x 2 x 2 mm, matrix = 256 x 256, FoV = 256 mm, number of sections = 64, slice thickness = 2 mm. 2 different b values (b = 0 and b = 1000 s / mm2) were used.

High resolution T1-weighted anatomic images were obtained with the following acquisition parameters: TE =3.02 ms, TR = 2600 ms, TI = 900 ms, flip angle = 8°, 176 slices of 1.0 mm thickness, matrix = 256 x 256, scan time of 7 minutes 18 seconds. The resulting voxel size was 1x 1x 1 mm.

3.2. MRI Preprocessing

For the anatomical reference and the definition of the network node, T1-weighted images were used. T1-weighted images were processed by using the recon-all pipeline of FreeSurfer (FreeSurfer 7.1.0; http://surfer.nmr.mgh.harvard.edu/). For performing the cortical reconstruction step, the T1-weighted image was aligned to MNI space. The skull was removed from the aligned T1-weighted image. Desikan-Killiany parcellations were transferred to T1-weighted space using the spherical surface warp created by FreeSurfer. Correction of the susceptibility-induced distortions, eddy current distortions, and motion artifacts for structural preprocessing on DWI data were executed by applying the FSL top-up and FSL eddy tools in FSL package (FSL 6.0; https://fsl.fmrib.ox.ac.uk/fsl/fslwiki) (Jenkinson et al., 2012). The white matter organization in

each voxel from the measured DWI data was estimated based on the peaks in the diffusion signal in voxels that emerged by restricting the movement of the water molecules through white matter fibers. Modelling the measured signal in a voxel was operated via the Diffusion Tensor Imaging (DTI) reconstruction method by a single tensor that defines the diffusion profile as one preferred diffusion direction per voxel. The informed RESTORE algorithm in the Connectivity Analysis TOolbox (CATO, v2.5) (de Lange et al., 2023) was used for the tensor estimation while identifying and removing the outliers during the fitting procedure. In this way, the effect of the physiological noise artifacts on the diffusion tensor modeling was decreased. Generalized Q-Sampling Imaging (GQI) calculates the diffusion signal. Then, the direction of the diffusion with the highest signal are selected as diffusion peaks, resulting in the detection of multiple peaks in a voxel. CATO toolbox was used for combining the DTI with a more advanced diffusion model, which is Generalized Q-Sampling Imaging (GQI), to acquire reconstruction of diffusion signal in multiple directions, resulting in a better depiction of the diffusion signal within voxels containing a more complex underlying white matter organization. Reconstruction of all possible white matter tracts between regions based on the diffusion peaks in each voxel was performed by the CATO implementation of the "Fiber Assignment by Continuous Tracking" (FACT) algorithm (Mori et al., 1999) with eight seed points per voxel, FA threshold of 0.1 and maximal angle of 55 degrees.

3.3. Anatomical Connectome Reconstruction

The following steps were applied to reconstruct the anatomical connectome by using the publicly available CATO toolbox:

For each participant, we obtained a network of 114 brain regions as well as reconstructed white matter streamlines between these brain regions. To this end, cortical reconstruction was processed in FreeSurfer for the preprocessing of the structural T1-weighted data consisting of parcellation of the cortex into 114 discrete regions based on the Cammoun subdivision of the Desikan-Killiany atlas (Cammoun et al., 2012). Summarized region properties of volumetric and brain surface statistics for each region were collected and used in later steps of the CATO pipeline.

To reconstruct the streamlines, deterministic streamline tractography based on the FACT algorithm was used since it gives a plausible balance between false-negative and false-positive reconstruction of fibers (Sarwar et al., 2019). The anatomical connectome of each participant was then stored in a connectivity matrix. Rows and columns of the matrix represent nodes, reflecting brain areas, while entries in the matrix represent edges, reflecting white matter fiber tracts. A 114 x 114 connectivity matrix detailing the whole reconstructed region-to-region connections was calculated for each participant. Connection weights included both streamline count (number of streamlines connecting two brain areas, NOS) and fractional anisotropy (FA) of voxels traversed by streamlines connecting two areas, which is commonly used as a marker of white matter microstructure. We focused on FA of reconstructed tractography because substantial heritability was observed in both twin MRI (Arnatkeviciute et al., 2021) and genome-wide association studies (Zhao et al., 2021), in addition to NOS as a measure of connection strength.

The structural and diffusion brain magnetic resonance imaging (MRI) were included in the structural connectome analysis. The incomplete or missing MRI, or participants whose structural

connectivity matrix included more than ten nodes having a rank of 0 were excluded and were not utilized for the further analysis (Levakov et al., 2021).

4. Statistical Analyses

In the main dataset, group differences were compared between high and low PLE groups using Mann-Whitney U test for age and Chi-square test for sex. In the external datasets, differences between FEP and HC groups were assessed using an independent samples t-test for age and a Chi-square test for sex.

5. Machine Learning Pipeline

Following the preprocessing steps, the Least Absolute Shrinkage and Selection Operator (LASSO) model was used on the train set. The primary purpose of LASSO regression is variable selection, achieved by setting the weights of unimportant variables to zero. Variables with non-zero coefficients were retained for the model. In the LASSO method, the lambda parameter, which is the weight parameter of the penalty term applied to the model coefficients, needs to be optimized. A grid search determined the optimal lambda parameter within a logarithmic range of 10^-3 to 10^3, minimizing model error. The alpha was set to 1 automatically. Subsequently, a classification model was developed using the Elastic Net algorithm, which combines LASSO and Ridge methods to optimize variable weights rather than solely focus on selection. Both alpha and lambda parameters were optimized by using the tuneLength argument in the elastic net method. This argument tried random parameter combinations and selected the most suitable combination. Thus, LASSO identified important variables, while the Elastic Net established the final classification model.

Although Elastic Net includes both LASSO and Ridge regularization, we applied GEE and LASSO in preliminary steps for the following reasons: First, GEE was used to consider the correlation structure, which is particularly crucial when the predictors involve clustered data. Since our data consists of siblings, the cluster structure was considered as families. Elastic Net and other machine-learning-based classification algorithms do not explicitly model such correlation structures. Second, LASSO was applied before Elastic Net as a variable selection procedure to reduce model complexity by eliminating unimportant features. These steps allowed us to focus Elastic Net modeling on a reduced, more informative set of predictors in the final step, enhancing stability and interpretability.

Data analysis was performed using R statistical software (version 4.3.0). “correlation” (Makowski et al., 2020), “gee” (Carey et al., 2012) and “caret” (Kuhn, 2008) R packages were used to conduct correlation, GEE, and ML analysis, respectively. “GMDH2” (Dag et al., 2019) R package was used to calculate performance measures. Accuracy, sensitivity, specificity, positive predictive value, negative predictive value, F1 score, and area under the ROC curve (AUC) were used to evaluate the classification performance. The seed number was specified as 123 to ensure repeatability. The scripts for the analysis in this study are publicly available in this GitHub repository: https://github.com/mervekasikci/ML_PP.

Table S1. Graph Metrices, Definitions and Interpretations

Metrices	Definition	Interpretations
Normalized Global Efficiency	Measures how easily information travels across the whole brain network by averaging the inverse of the shortest path lengths between regions.	Indication of how efficiently information can be exchanged across the whole network. Corrected for differences in numbers of edges between participants.
Density	The proportion of actual connections in the network compared to all potential connections.	Indication of how "wired" the brain network is.
Normalized Local Efficiency	Local information processing capacity of a network	Indication of how efficiently information is exchanged between nearby neighbors of a given network node
Normalized Betweenness Centrality	Measures how often a brain region serves as a bridge for communication between other regions by being part of the shortest paths.	Indication of how important a region is for relaying information. Corrected for differences in numbers of edges between participants.
Normalized Clustering Coefficient	Average probability of a node's tendency to cluster with its neighbors	Indication of functional segregation. Corrected for differences in numbers of edges between participants.

6. Results

Table S2. Distribution of Psychotic-like Experiences (PLEs) by Zygosity

**Zygosity**	**Low PLEs, *n (%)***	**High PLEs, *n (%)***	**Total, *n (%)***
DZ/Siblings	126 (63)	123 (62.8)	249 (62.9)
MZ	71 (35.5)	70 (35.7)	141(35.6)
Triplets	3 (1.5)	3 (1.5)	6 (1.5)

Total	200 (50.5)	196 (49.5)	396 (100)

Abbreviations: DZ = dizygotic twins; MZ = monozgotic twins.

Table S3. Summary Statistics of Psychotic-like Experiences Sample

Variables	Psychotic-like Experiences Sample (*N* =396)	Minimum	Maximum
Parental Social Class			
Low, *n (%)*	129 (32.58)		
Middle, *n (%)*	192 (48.48)		
High, *n (%)*	73 (18.43)		
Missing, *n (%)*	2 (0.51)		
Environmental Risk Score (ERS), *mean (SD)*[a]	0.45 (2.48)	-4.5	10.5
Threatening Life Experiences, *mean (SD)*	2.43 (1.94)	0	9
Social Cohesion and Trust, *mean (SD)*	30.79 (8.22)	3	55
Informal Social Control, mean (SD)	5.37 (3.10)	0	17
Discrimination, *mean (SD)*	1.22 (1.47)	0	8
Psychotic-like Experiences, *mean (SD)*	74.33 (16.21)	42	133
Negative-self, *mean (SD)*	4.77 (4.5)	0	22
Negative-others, *mean (SD)*	6.48 (4.75)	0	24
Positive-self, *mean (SD)*	13.55 (6.13)	0	24
Positive-others, *mean (SD)*	8.51 (4.21)	0	24
Psychiatric Disorders in First-degree Relatives			
Present, *n (%)*	140 (35.35)		
Absent, *n (%)*	254 (64.14)		
Missing, *n (%)*	2 (0.51)		

Abbreviation: SD = standard deviation.

[a] Negative values denote lower cumulative environmental risk, positive values denote elevated cumulative environmental risk, and 0 reflects average exposure to risk factors.

6.1. Marginal Model (Generalized Estimating Equations (GEE)

Variables identified as significant predictors (explanatory variables) of psychotic-like experiences based on the results of the GEE analysis, full list of significant predictors, ORs and 95% CIs are presented in Supplementary Table 4.

Table S4. Significant Variables from Generalized Estimating Equations to Predict Psychotic-like Experiences

**Environmental Risk Factors**	**OR**	**95%LL**	**95%UL**	**p-value**
Parental social class (middle)	1.703	1.03	2.83	0.040
Total Discrimination	1.260	1.1	1.45	<0.001
Total score of threatening life events	1.209	1.085	1.349	0.001
Informal social control	1.108	1.04	1.19	0.003
Social cohesion and trust	0.961	0.94	0.99	0.002
Total score for childhood trauma	1.069	1.04	1.10	<0.001
Emotional abuse	1.258	1.15	1.37	<0.001
Physical neglect	1.164	1.05	1.29	0.005
Emotional neglect	1.112	1.06	1.17	<0.001
Environmental risk score for psychosis	1.200	1.10	1.31	<0.001
Clinical Risk Factor				
History of psychiatric disorders in first degree relatives	1.840	1.17	2.9	0.008
Cognitive Appraisals				
Negative-self	1.242	1.16	1.33	<0.001
Positive-self	0.951	0.92	0.99	0.006
Negative-others	1.160	1.10	1.22	<0.001
Positive-others	0.92	0.88	0.97	0.001
**Covariates**	**OR**	**95%LL**	**95%UL**	**p-value**
Age	1.021	0.937	1.113	0.632
Sex	0.878	0.578	1.332	0.540
PRI composite score	1.011	0.996	1.026	0.159
**Brain Network Indices (NOS-weighted)**	**OR**	**95%LL**	**95%UL**	**p-value**

Local efficiency in the left fusiform 1	447363	1.69	1.19×10^{11}	0.041
Local efficiency in the left inferior temporal 1	0.000	0.000	0.154	0.011
Local efficiency in the right inferior temporal 1	0.000	0.000	0.505	0.033
Local efficiency in the right lateral orbitofrontal 2	0.000	0.000	0.166	0.023
Local efficiency in the right medial orbitofrontal 1	0.000	0.000	0.479	0.039
Betweenness centrality in the left precuneus 1	7.457	1.207	46.057	0.031
Betweenness centrality in the right lateral occipital 1	436502	180	1.06×10^{9}	0.001
Betweenness centrality in the right pericalcarine 1	0.005	0.00	0.730	0.037
Betweenness centrality in the right rostral middle frontal 1	36.83	1.59	851.6	0.024
Betweenness centrality in the right superiorfrontal 2	21.91	2.32	207.18	0.007
Clustering coefficient in the left inferior temporal 1	0.000	0.000	0.107	0.010
Clustering coefficient in the left entorhinal 1	0.000	0.000	0.883	0.048
Clustering coefficient in the right inferior temporal 1	0.000	0.000	0.605	0.040
Clustering coefficient in the left middle temporal 1	0.000	0.000	0.302	0.032
Clustering coefficient in the right lateral orbitofrontal 2	0.000	0.000	0.099	0.022
Clustering coefficient in the right medial orbitofrontal 1	0.000	0.000	0.733	0.046
**Brain Network Indices (FA-weighted)**	**OR**	**95%LL**	**95%UL**	**p-value**
Local efficiency in the left fusiform 2	8.313	1.093	63.215	0.041
local efficiency in the left precuneus 2	19.09	1.165	312.95	0.039
Local efficiency in the right superior temporal 2	6.357	1.003	40.294	0.050
Betweenness centrality in the left superior frontal 1	0.000	0.000	0.575	0.038
Betweenness centrality in the left superior parietal 3	0.007	0.000	0.394	0.016

Clustering coefficient in the left fusiform 2	19.32	1.151		324.29	0.040
Clustering coefficient in the left pericalcarine 1	7.406	1.033		53.114	0.046
Clustering coefficient in the left superior temporal 2	45.91	1.242		1697.2	0.038
Clustering coefficient in the right superior temporal 2	12.613	1.098		144.91	0.042
Clustering coefficient in the right insula 1	21.084	1.163		382.25	0.039

Note. Zygosity was included in the GEE model with monozygotic twins as the reference category. Dizygotic twins and siblings (OR = 0.976, 95% CI = 0.616-1.547, p = 0.917 and triplets (OR = 1.018, 95% CI = 0.377-2.749, p = 0.972) showed no significant associations with psychotic-like experiences.

Abbreviations: FA = fractional anisotropy; LL = lower limit; NOS = number of streamlines; OR = odds ratio; PRI = Perceptual Reasoning Index; UL = upper limit.

6.2. Correlation Filter

The correlations between the explanatory variables were examined, and highly correlated (r>0.70) pairs of variables were identified. **34** explanatory variables remained. Accordingly, extracted variables and their statistical results are presented in Supplementary Table 5.

Table S5. Correlations of Model Variables to Predict Psychotic-like Experiences

**Variable 1**	**Variable 2**	**Spearman rho**	**CI_LL**	**CI_UL**	**p-value**
Local Efficiency (NOS-weighted)	Clustering Coefficient (NOS-weighted)				
left inferior temporal 1	left inferior temporal 1	0.989	0.986	0.991	<0.001
right inferior temporal 1	right inferior temporal 1	0.988	0.986	0.991	<0.001

right lateral orbitofrontal 2	right lateral orbitofrontal 2	0.984	0.98	0.987	<0.001
right medial orbitofrontal 1	right medial orbitofrontal 1	0.971	0.965	0.977	<0.001
Local Efficiency (FA-weighted)	Clustering Coefficient (FA-weighted)				
left fusiform 2	left fusiform 2	0.896	0.874	0.915	<0.001
right superior temporal 2	right superior temporal 2	0.886	0.861	0.906	<0.001

Abbreviations: CI = confidence interval; FA = fractional anisotropy; LL = lower limit; NOS = number of streamlines; UL = upper limit.

6.3. Data Preprocessing

During the dummy variable coding, among the 34 variables, parental social class was a categorical variable with more than 2 categories. Dummy variable coding was applied to prepare parental social class variable for further analysis. One category was used as the reference category and the other categories were converted into binary (0 or 1) variables. Since parental social class has three categories, one of them was taken as a reference (high level) and the two dummy variables were generated for the other categories (low and middle). Thus, the number of explanatory variables increased from 34 to 35. Of the initial 396 participants, 20 were excluded because of missing values on selected predictors. Therefore, the dataset included 376 participants with complete data across the selected variables. Specifically, 6 participants with missing PRI composite score, 2 participants with missing parental social class, 8 participants with missing social cohesion and trust scores, 2 participants with total discrimination score, 1 participant with missing cognitive appraisal scores and 1 participant with missing betweenness centrality measure in left precuneus were excluded. The dataset was divided into 70% train set and 30% test set. Z-transformation was

applied to the train set to obtain standardized coefficients from the models. The test set was also standardized based on the characteristics of the train set.

6.4 Feature Selection with LASSO

The variables whose coefficients were shrunk to 0 through LASSO and, thus, excluded in this step were family history of psychiatric disorders in the first-degree relatives, local efficiency (NOS-weighted) in the brain area of the right medial orbitofrontal 1, local efficiency (FA-weighted) in the brain area of the left fusiform 2 and right superior temporal 2, clustering coefficient (FA-weighted) in the brain area of the left superior temporal 2. The variables remained in the model is presented in Supplementary Table 6. Consequently, **30** explanatory variables remained. After completing the variable selection, the next step was developing the classification model.

6.5 Directionality of the Predictors

SHAP explainer method provided more detailed evaluation of the effects of the predictors (Figure S1).

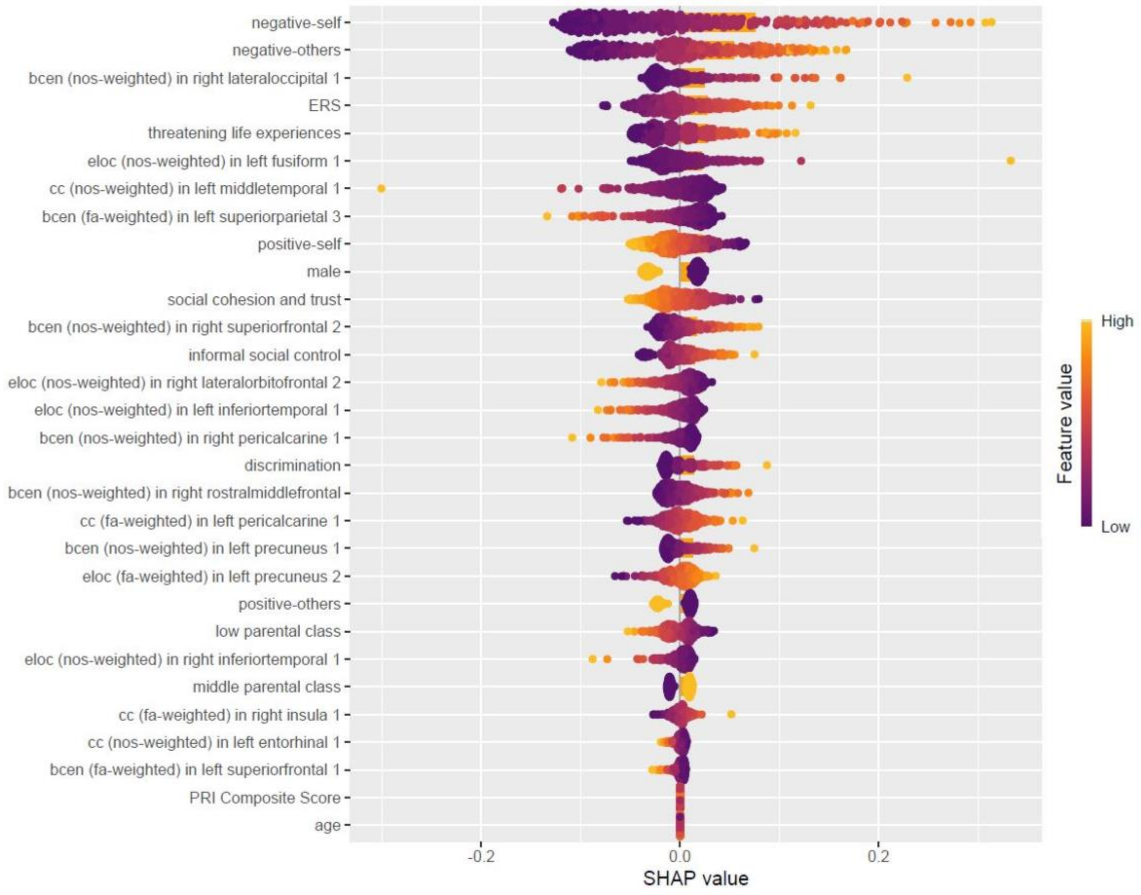

Figure S1. *Directionality of predictors based on SHAP values in Elastic Net model predictions.* This plot illustrates the impact of predictors on the prediction of the Elastic Net model. Each point represents an individual participant's data, with color indicating feature values (yellow = high, purple = low). A positive SHAP score implies that the feature elevates the prediction (toward the positive class), whereas a negative value lowers it (toward the negative class). Abbreviations: bcen = betweenness centrality; cc = clustering coefficient; eloc = local efficiency; ERS = environmental risk score for psychosis; FA = fractional anisotropy; NOS = number of streamlines; PRI = perceptual reasoning index.

The purple color represented the low value of the predictor, and the yellow color represented the high value. If the SHAP value is negative, that predictor pulls the model's prediction toward the negative class (low PLE). If the SHAP value is positive, that predictor pulls the model's prediction toward the positive class (high PLE). For example, as shown in Figure 3, the variables negative-self, negative-others, and ERS exhibit a pattern where negative SHAP values (on the x-axis) are predominantly represented by purple-colored points, while positive SHAP values are

mainly depicted by yellow-colored points. This indicates that lower variable values are associated with predictions toward the low PLE, whereas higher variable values correspond to predictions toward the high PLE. In contrast, for the positive-self and positive-others variables, negative SHAP values are predominantly represented by yellow-colored points, while positive SHAP values are represented mainly by purple-colored points. This indicates that lower variable values are associated with predictions of high PLE, while higher variable values correspond to predictions of low PLE.

Table S6. Variables Remained in the Model

**1**	age	**2**	PRI composite score
**3**	Discrimination	**4**	Threatening life events
**5**	Informal Social Control	**6**	Social Cohesion and Trust
**7**	Negative-self	**8**	Positive-self
**9**	Negative others	**10**	Positive-others
**11**	Environmental risk score (ERS)	**12**	local efficiency in left fusiform 1 (nos-weighted)
**13**	local efficiency in left inferior temporal 1 (nos-weighted)	**14**	local efficiency in right inferior temporal 1 (nos-weighted)
**15**	local efficiency in right lateral orbitofrontal 2 (nos-weighted)	**16**	betweenness centrality in right lateral occipital 1 (nos-weighted)
**17**	betweenness centrality in left precuneus 1 (nos-weighted)	**18**	betweenness centrality in right rostral middle frontal 1 (nos-weighted)

**19**	betweenness centrality in right pericalcarine 1 (nos-weighted)	**20**	clustering coefficient in left entorhinal 1 (nos-weighted)
**21**	betweenness centrality in right superior frontal 2 (nos-weighted)	**22**	betweenness centrality in left superior parietal 3 (fa-weighted)
**23**	clustering coefficient in left middle temporal 1 (nos-weighted)	**24**	sex
**25**	local efficiency in left precuneus 2 (fa-weighted)	**26**	Parental social class (middle)
**27**	betweenness centrality in left superior frontal 1 (fa-weighted)		
**28**	clustering coefficient in left pericalcarine 1 (fa-weighted)		
**29**	clustering coefficient in right insula 1 (fa-weighted)		
**30**	Parental social class (low)		

Notes. Variables were selected using least absolute shrinkage and selection operator (LASSO) regression. The numbers in the table do not indicate ranking or order of significance; they are for reference only. Abbreviations: ERS = environmental risk score for psychosis; FA = fractional anisotropy; NOS = number of streamlines; PRI = perceptual reasoning index.

Table S7. Sample Characteristics of First Episode of Psychosis Patients, Their Siblings, and Healthy Control Group

**Variables**	**FEP Patients (n = 16)**	**Siblings (n =19)**	**Controls (n = 18)**
Sex			
Females, *n (%)*	7 (43.75)	10 (52.63)	7 (38.9)
Males, *n (%)*	9 (56.25)	9 (47.37)	11 (61.1)
Age, *mean (SD)*	19.56 (3.14)	18.21 (3.77)	19.44 (2.09)
Parental Social Class			
Low, *n (%)*	13 (81.25)	16 (84.21)	17 (94.4)
Middle, *n (%)*	3 (18.75)	2 (10.53)	1 (5.6)
High, *n (%)*	0	1 (5.26)	0
PRI Composite Score	81.38 (17.26)	90.58 (14.66)	81.67 (14.36)
ERS, *mean (SD)*	0.59 (0.83)	-1 (2.35)	-1.39 (1.91)
Threatening Life Experiences	2.75 (1.91)	1.95 (1.75)	1.56 (1.34)
Social Cohesion and Trust	31.94 (7.97)	31.05 (12.41)	30.67 (3.04)
Informal Social Control	7.13 (3.83)	5.84 (3.79)	5.39 (3.42)
Discrimination	1.94 (2.29)	1.11 (2.08)	0.61 (0.78)
Negative-self[a]	6.31 (6.42)	2.05 (1.75)	1.61 (2.75)
Negative-others[a]	7.25 (5.88)	6.06 (6.69)	2.94 (3.54)
Positive-self[a]	7.31 (5.72)	12.53 (7.00)	13.56 (7.37)

Positive-others[a]	5.38 (4.21)	10.11 (5.41)	11.56 (4.95)
Medication Duration/days, *mean (SD)*	156.55 (109.32)	NA	NA
Psychiatric Disorder in First-degree Relatives			
Present, *n (%)*	10 (62.5)	NA	13 (72.2)
Absent, *n (%)*	6 (37.5)	NA	5 (27.8)

Abbreviations: SD = standard deviation; NA = not applicable.

[a] Obtained using Brief Core Schema Scale (BCSS) where higher scores show stronger beliefs in each category. Higher negative-self or negative-others scores reflect more critical or mistrustful schemas, while higher positive-self or positive-others scores show more affirming and trusting schemas.

Table S8. Descriptive Statistics of Structural Network Properties of External Validation Cohort

	**Siblings**			**FEP Patients**			**Healthy Controls**		
**Variables**	**Mean**	**N**	**Standard Deviation**	**Mean**	**N**	**Standard Deviation**	**Mean**	**N**	**Standard Deviation**
local efficiency in left fusiform 1 (nos-weighted)	0.023	19	0.016	0.027	16	0.014	0.022	18	0.011
local efficiency in left inferior temporal 1 (nos-weighted)	0.028	19	0.011	0.035	16	0.020	0.028	18	0.011
local efficiency in right inferior temporal 1 (nos-weighted)	0.029	19	0.014	0.029	16	0.016	0.029	18	0.014
local efficiency in right lateral orbitofrontal 2 (nos-weighted)	0.024	19	0.020	0.023	16	0.018	0.025	18	0.017

local efficiency in right medial orbitofrontal 1 (nos-weighted)	0.017	19	0.019	0.016	16	0.017	0.015	18	0.016
betweenness centrality in left precuneus 1 (nos-weighted)	0.042	19	0.049	0.070	16	0.126	0.034	18	0.048
betweenness centrality in right lateral occipital 1 (nos-weighted)	0.023	19	0.022	0.018	16	0.017	0.026	18	0.022
betweenness centrality in right pericalcarine 1 (nos-weighted)	0.058	19	0.093	0.024	16	0.024	0.031	18	0.031
betweenness centrality in right rostral middle frontal 1 (nos-weighted)	0.109	19	0.079	0.074	16	0.053	0.090	18	0.081
betweenness centrality in right superior frontal 2 (nos-weighted)	0.128	19	0.106	0.062	16	0.071	0.128	18	0.107
clustering coefficient in left enthorhinal 1 (nos-weighted)	0.012	19	0.010	0.016	16	0.015	0.013	18	0.014
clustering coefficient in left middle temporal 1 (nos-weighted)	0.022	19	0.011	0.018	16	0.012	0.019	18	0.010
local efficiency in left fusiform 2 (fa-weighted)	0.361	19	0.103	0.375	16	0.068	0.357	18	0.090

local efficiency in left precuneus 2 (fa-weighted)	0.388	19	0.081	0.368	16	0.074	0.371	18	0.074
local efficiency in right superior temporal 2 (fa-weighted)	0.429	19	0.075	0.425	16	0.078	0.418	18	0.072
betweenness centrality in left superior frontal 1 (fa-weighted)	0.020	19	0.015	0.031	16	0.033	0.025	18	0.018
betweenness centrality in left superior parietal 3 (fa-weighted)	0.032	19	0.028	0.025	16	0.029	0.027	18	0.030
clustering coefficient in left pericalcarine 1 (fa-weighted)	0.306	19	0.086	0.295	16	0.081	0.316	18	0.079
clustering coefficient in left superior temporal 2 (fa-weighted)	0.229	19	0.052	0.235	16	0.047	0.222	18	0.046
clustering coefficient in right insula 1 (fa-weighted)	0.227	19	0.069	0.235	16	0.055	0.210	18	0.066

Abbreviations: FA = fractional anisotropy; NOS = number of streamlines.

Table S9. Confusion Matrix and Performance Metrics of Patient-Healthy Controls

**Metric**				
		**Class[a]**		
**Confusion Matrix**	**Predicted Class**	**Patient**	**Control**	**Total**
	Patient	6 (TP)	10 (FP)	16
	Control	10 (FN)	8 (TN)	18
	Total	16	18	34

Performance Metrics	Value
Accuracy	0.412
Sensitivity	0.375
Specificity	0.444
Positive Predictive Value	0.375
Negative Predictive Value	0.444
F1	0.375
AUC (95% C.I.)	0.590 (0.420-0.760)

Abbreviations: AUC = area under the receiver operating characteristic curve; CI = confidence interval; FN = false negative; FP = false positive; TN = true negative; TP = true positive.
[a]Patient class included FEP patients while control class included healthy control participants.

REFERENCES

Arnatkeviciute, A., Fulcher, B. D., Bellgrove, M. A., & Fornito, A. (2021). Where the genome meets the connectome: Understanding how genes shape human brain connectivity. In *NeuroImage* (Vol. 244). https://doi.org/10.1016/j.neuroimage.2021.118570

Benton, A. L. (1968). Differential behavioral effects in frontal lobe disease. *Neuropsychologia*, *6*(1). https://doi.org/10.1016/0028-3932(68)90038-9

Bernstein, D. P., Stein, J. A., Newcomb, M. D., Walker, E., Pogge, D., Ahluvalia, T., Stokes, J., Handelsman, L., Medrano, M., Desmond, D., & Zule, W. (2003). Development and validation of a brief screening version of the Childhood Trauma Questionnaire. *Child Abuse and Neglect*, *27*(2). https://doi.org/10.1016/S0145-2134(02)00541-0

Borkowski, J. G., Benton, A. L., & Spreen, O. (1967). Word fluency and brain damage. *Neuropsychologia*, *5*(2). https://doi.org/10.1016/0028-3932(67)90015-2

Brugha, T. S., & Cragg, D. (1990). The List of Threatening Experiences: the reliability and validity of a brief life events questionnaire. *Acta Psychiatrica Scandinavica*, *82*(1). https://doi.org/10.1111/j.1600-0447.1990.tb01360.x

Cammoun, L., Gigandet, X., Meskaldji, D., Thiran, J. P., Sporns, O., Do, K. Q., Maeder, P., Meuli, R., & Hagmann, P. (2012). Mapping the human connectome at multiple scales with diffusion spectrum MRI. *Journal of Neuroscience Methods*, *203*(2). https://doi.org/10.1016/j.jneumeth.2011.09.031

Carey, V. J., Lumley, T., & Ripley, B. D. (2012). gee: Generalized estimation equation solver. In *UR L http://CRAN. R-project. org/package= gee, R package version*.

Dag, O., Karabulut, E., & Alpar, R. (2019). GMDH2: Binary classification via GMDH-type neural network algorithms—R package and web-based tool. *International Journal of Computational Intelligence Systems*, *12*(2). https://doi.org/10.2991/ijcis.d.190618.001

de Lange, S. C., Helwegen, K., & van den Heuvel, M. P. (2023). Structural and functional connectivity reconstruction with CATO - A Connectivity Analysis TOolbox. *NeuroImage*, *273*. https://doi.org/10.1016/j.neuroimage.2023.120108

Di Forti, M., Morgan, C., Dazzan, P., Pariante, C., Mondelli, V., Marques, T. R., Handley, R., Luzi, S., Russo, M., Paparelli, A., Butt, A., Stilo, S. A., Wiffen, B., Powell, J., & Murray, R. M. (2009). High-potency cannabis and the risk of psychosis. *British Journal of Psychiatry*, *195*(6). https://doi.org/10.1192/bjp.bp.109.064220

Gabbidon, J., Farrelly, S., Hatch, S. L., Henderson, C., Williams, P., Bhugra, D., Dockery, L., Lassman, F., Thornicroft, G., & Clement, S. (2014). Discrimination attributed to mental illness or race-ethnicity by users of community psychiatric services. *Psychiatric Services*, *65*(11). https://doi.org/10.1176/appi.ps.201300302

Jenkinson, M., Beckmann, C. F., Behrens, T. E. J., Woolrich, M. W., & Smith, S. M. (2012). Review FSL. *NeuroImage*, *62*.

Kirkbride, J. B., Boydell, J., Ploubidis, G. B., Morgan, C., Dazzan, P., McKenzie, K., Murray, R. M., & Jones, P. B. (2008). Testing the association between the incidence of schizophrenia and social capital in an urban area. *Psychological Medicine*, *38*(8). https://doi.org/10.1017/S0033291707002085

Kuhn, M. (2008). Building predictive models in R using the caret package. *Journal of Statistical Software*, *28*(5). https://doi.org/10.18637/jss.v028.i05

Levakov, G., Faskowitz, J., Avidan, G., & Sporns, O. (2021). Mapping individual differences across brain network structure to function and behavior with connectome embedding. *NeuroImage*, *242*. https://doi.org/10.1016/j.neuroimage.2021.118469

Makowski, D., Ben-Shachar, M., Patil, I., & Lüdecke, D. (2020). Methods and Algorithms for Correlation Analysis in R. *Journal of Open Source Software*, *5*(51). https://doi.org/10.21105/joss.02306

Mallet, R. (1997). Sociodemographic schedule. In *Section of Social Psychiatry, Institute of Psychiatry: London, UK*.

Mori, S., Crain, B. J., Chacko, V. P., & Van Zijl, P. C. M. (1999). Three-dimensional tracking of axonal projections in the brain by magnetic resonance imaging. *Annals of Neurology*, *45*(2). https://doi.org/10.1002/1531-8249(199902)45:2<265::AID-ANA21>3.0.CO;2-3

Newcombe, F. (1969). *Missile wounds of the brain: A study of psychological deficits.* Oxford U. Press.

Preti, A., Pisano, A., Cascio, M. T., Monzani, E., Meneghelli, A., & Cocchi, A. (2012). Obstetric complications in early psychosis: Relation with family history of psychosis. *Psychiatry Research*, *200*(2–3). https://doi.org/10.1016/j.psychres.2012.07.013

Sánchez-Gutiérrez, T., Rodríguez-Toscano, E., Roldán, L., Ferraro, L., Parellada, M., Calvo, A., López, G., Rapado-Castro, M., La Barbera, D., La Cascia, C., Tripoli, G., Di Forti, M., Murray, R. M., Quattrone, D., Morgan, C., Van Os, J., García-Portilla, P., Al-Halabí, S., Bobes, J., … Arango, C. (2023). Tobacco use in first-episode psychosis, a multinational EU-GEI study. *Psychological Medicine*, *53*(15). https://doi.org/10.1017/S0033291723000806

Sarwar, T., Ramamohanarao, K., & Zalesky, A. (2019). Mapping connectomes with diffusion MRI: deterministic or probabilistic tractography? *Magnetic Resonance in Medicine*, *81*(2). https://doi.org/10.1002/mrm.27471

Shao, Z., Janse, E., Visser, K., & Meyer, A. S. (2014). What do verbal fluency tasks measure? Predictors of verbal fluency performance in older adults. *Frontiers in Psychology*, *5*(JUL). https://doi.org/10.3389/fpsyg.2014.00772

Vassos, E., Sham, P., Kempton, M., Trotta, A., Stilo, S. A., Gayer-Anderson, C., Di Forti, M., Lewis, C. M., Murray, R. M., & Morgan, C. (2020). The Maudsley environmental risk score for psychosis. *Psychological Medicine*, *50*(13). https://doi.org/10.1017/S0033291719002319

Wechsler, D. (2011). *Wechsler Abbreviated Scale of Intelligence--Second Edition (WASI-II)* (Second). Pearson. https://doi.org/https://doi.org/10.1037/t15171-000

Zhao, B., Zhang, J., Ibrahim, J. G., Luo, T., Santelli, R. C., Li, Y., Li, T., Shan, Y., Zhu, Z., Zhou, F., Liao, H., Nichols, T. E., & Zhu, H. (2021). Large-scale GWAS reveals genetic architecture of brain white matter microstructure and genetic overlap with cognitive and mental health traits (n = 17,706). *Molecular Psychiatry*, *26*(8). https://doi.org/10.1038/s41380-019-0569-z
